# Risk factors in HIV/AIDS patients with cryptococcal meningitis

**DOI:** 10.1186/1471-2334-14-S4-P29

**Published:** 2014-05-29

**Authors:** Ioana Bădicuț, Simona Paraschiv, Alina Borcan, Daniela Tălăpan, Olga Dorobăț

**Affiliations:** 1 National Institute for Infectious Diseases “Prof. Dr. Matei Balş”, Bucharest, Romania

## 

Cryptococcosis represents a major life-threatening fungal infection in patients with severe HIV infection. Cryptococcal meningoencephalitis is the most common manifestation of cryptococcosis in patients with advanced immunosuppression. Objectives: evaluating risk factors for severe evolution of patients with HIV infection and meningitis.

Possible risk factors were analyzed in 33 HIV-infected patients with meningitis, of the 2,100 patients monitored for HIV infection in the National Institute for Infectious Diseases “Prof. Dr. Matei Balş” during 2011-2013. Epidemiological data, CD4 serum and glycorrhachia values were collected from patient records. Etiological diagnosis was made by direct microscopic examination, stained smears microscopy, India ink test, cryptococcus antigen identification by latex agglutination and culture. Identification and antifungal susceptibility testing were performed with Vitek 2C analyzer. HIV-1 genotyping was performed in protease (PR) and partial reverse transcriptase (RT) regions by using DNA sequencing and Viroseq™ HIV-1 Genotyping System. For subtyping purposes the sequences were analyzed with REGA HIV-1&2 automated subtyping tool version 2.0.

A number of 12 patients were found positive for Latex agglutination cryptococcus test. Half of the patients were parenterally infected children in the late ’80s and the rest were sexually infected. All patients were in immunological suppression of C3 stage and without therapy on admission. The fatal evolution was associated with tuberculosis (TB, 4 cases) and HBV infection (1 case). CD4 serum values ranged from 42 cells/cmm (7 patients alive) to 22 cells/cmm (5 dead). Cerebrospinal fluid glucose was 33 mg/dL in the 7 alive patients and 23.6 mg/dL in the 5 deceased. All samples having positive latex, had India Ink positive. Only three samples showed no growth in culture. The analyzed patients were infected with subtype F1 HIV-1 viruses, as indicated by subtyping analysis. All the patients had antiretroviral (ARV) therapy interrupted at the moment of cryptococcal meningitis diagnosis. As expected, resistance mutations were not present in PR and RT genes, with few exceptions such as K103N, E138A.

The cryptococcal meningitis is very rare among Romanian HIV-1 infected patients, frequently associated with advanced immune suppression, lack of ARV therapy and other comorbidities (TB and HBV infection).

